# Supplementary material for: BMPR2 Variants Underlie Nonsyndromic Oligodontia
Source: Int J Mol Sci. 2023 Jan 13;24(2):1648. doi: 10.3390/ijms24021648 (PMC9860601; doi:10.3390/ijms24021648)
Supplement: Supplementary file 1 [file ijms-24-01648-s001.zip › ijms-2039845-supplementary.pdf]

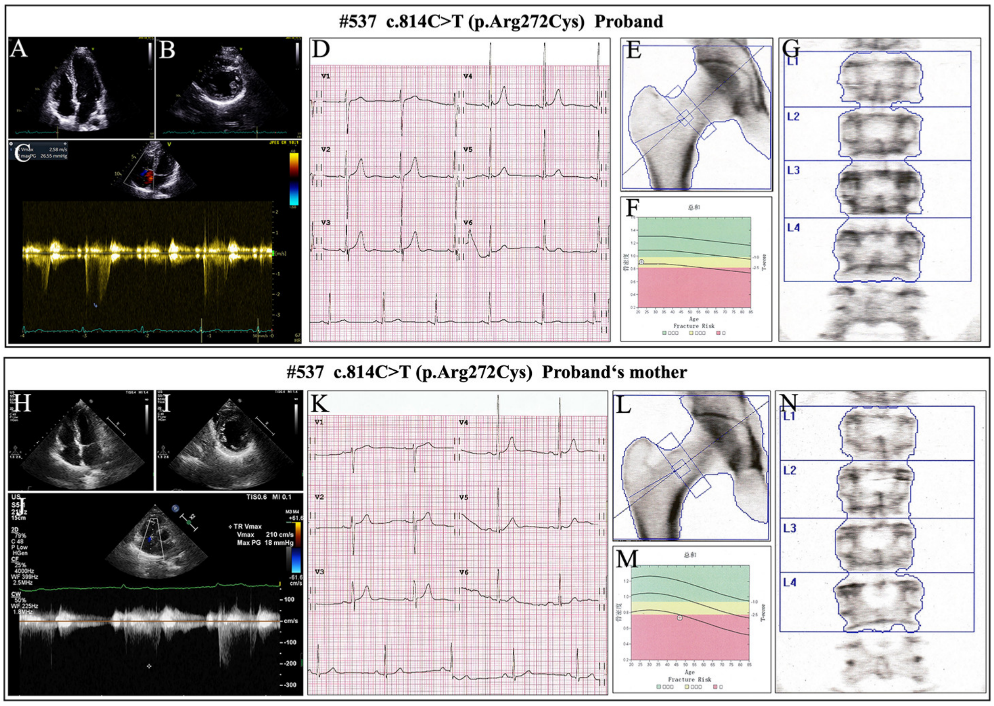

Figure S1. Echocardiographic, electrocardiographic, and DEXA examination of family #537. (A–C) Echocardiographic images of #537 proband carrying a *BMPR2* variant, c.814C>T (p.Arg272Cys); (D) Electrocardiographic examination result of #537 proband; (E–G) DEXA scan images of #537 proband; (H–J) Echocardiographic images of the mother of #537 proband carrying a *BMPR2* variant, c.814C>T (p.Arg272Cys); (K) Electrocardiographic examination of the mother of #537 proband; (L–N) DEXA scan images of the mother of #537 proband. DEXA, dual-energy X-ray absorptiometry; *BMPR2*, bone morphogenetic protein receptor type 2.
